# *TSPmap*, a tool making use of traveling salesperson problem solvers in the efficient and accurate construction of high-density genetic linkage maps

**DOI:** 10.1186/s13040-017-0158-0

**Published:** 2017-12-19

**Authors:** J. Grey Monroe, Zachariah A. Allen, Paul Tanger, Jack L. Mullen, John T. Lovell, Brook T. Moyers, Darrell Whitley, John K. McKay

**Affiliations:** 1 Department of Bioagricultural Sciences & Pest Management, Colorado State University, 1177 Campus Delivery, Fort Collins, CO 80523 USA; 2 Department of Computer Sciences, Colorado State University, Fort Collins, CO 80523 USA; 3 Genome Sequencing Center, HudsonAlpha Institute for Biotechnology, Huntsville, AL 35806 USA

**Keywords:** Genetic mapping, Linkage, Travelling salesperson problem, Genomic markers, Next generation sequencing, Genotyping by sequencing

## Abstract

**Background:**

Recent advances in nucleic acid sequencing technologies have led to a dramatic increase in the number of markers available to generate genetic linkage maps. This increased marker density can be used to improve genome assemblies as well as add much needed resolution for loci controlling variation in ecologically and agriculturally important traits. However, traditional genetic map construction methods from these large marker datasets can be computationally prohibitive and highly error prone.

**Results:**

We present *TSPmap*, a method which implements both approximate and exact Traveling Salesperson Problem solvers to generate linkage maps. We demonstrate that for datasets with large numbers of genomic markers (e.g. 10,000) and in multiple population types generated from inbred parents, *TSPmap* can rapidly produce high quality linkage maps with low sensitivity to missing and erroneous genotyping data compared to two other benchmark methods, *JoinMap* and *MSTmap*. *TSPmap* is open source and freely available as an R package.

**Conclusions:**

With the advancement of low cost sequencing technologies, the number of markers used in the generation of genetic maps is expected to continue to rise. *TSPmap* will be a useful tool to handle such large datasets into the future, quickly producing high quality maps using a large number of genomic markers.

**Electronic supplementary material:**

The online version of this article (10.1186/s13040-017-0158-0) contains supplementary material, which is available to authorized users.

## Background

Genetic maps are the foundation of genotype to phenotype mapping and a critical component in the discovery of the molecular basis of both simple and complex traits. Increased sample size and number of markers in a map improves the resolution of chromosomal regions underlying quantitative trait loci (QTL) and reduces the number of possibly causal variants for further investigation. Additionally, genetic maps are a valuable tool for constraining and validating the assembly of eukaryotic genomes because genetic linkage map construction is robust to repetitive regions and paralogs, which can confound assembly algorithms based purely on sequence data. These motivations, and recent advances in genotyping by sequencing (GBS) technologies, have led to dramatic increases in the number of markers used to generate genetic maps. Indeed, recent reports demonstrate the use of maps composed of more than 10,000 markers [1–5].

While next generation sequencing technologies have allowed for the identification of increasing number of genetic markers, the rate of erroneous calls, on the order of 1–2% [6–8], affect map quality. The often-high rates of missing genotype data resulting from popular GBS methods also can create problems for mapping algorithms. The increasing risk of mis-clustering and mis-ordering of markers with increasing size of datasets demands computational methods that can efficiently generate genetic maps with larger marker datasets while effectively handling missing or erroneous marker calls.

To date, software methods for generating genetic maps have used strategies such as simulated annealing to maximize a likelihood function [9–12], graphical approaches such as minimum spanning tree of the marker graph [13] or projecting a principal coordinate analysis onto a 3D trend line [14]. Since the late 1990s it has been recognized that genetic linkage mapping could be conceptualized as a Travelling Salesperson Problem (TSP) [11, 15–17]. However the application of TSP solvers has yet to be implemented into a usable open source genetic mapping tool.

The TSP is formulated as a problem in which an agent wishes to visit all of the vertices of a graph, G(V,E). The edges of the graph are weighted. The goal is to find a Hamiltonian circuit (a “tour”) that visits all of the vertices using *n* edges, and that also results in the lowest cost, meaning that the sum of the weighted edges on the Hamiltonian circuit is minimized. Each genetic marker corresponds to a vertex in the graph G(E,V), and the recombination frequency (*rf*) values between markers represent the weights between the vertices. This allows the recombination frequency matrix to serve as the weight matrix for the TSP instance, and the solution will give us the lowest-cost path through the markers. However, no genetic mapping tool using an exact TSP solver has been developed to date. This is likely because only recently has the computational power required to implement exact TSP solvers for large datasets been achieved for personal computers, finally unlocking the potential to apply this approach toward generating genetic maps [18].

Another computational framework that can be applied to the problem of finding genetic linkage maps is the *minimal spanning tree* (MST). Finding the minimal spanning tree of a graph, G(V,E), has a polynomial time complexity of O(V + E). By contrast, the TSP is an NP-Hard problem, meaning there is no known deterministic algorithm for solving all TSP instances in polynomial time. Thus it would seem better to use a minimal spanning tree if possible to generate genetic linkage maps. Under perfect conditions, one can indeed use a minimal spanning tree to generate accurate genetic linkage maps. By perfect conditions, we mean that the recombination frequencies exactly and precisely capture the distance between genetic markers. This means, for example, if *m1, m2, m3, m4, m5* are genetic markers that are already in the correct order, then the distance between *m3* and *m4* must be less than the distance between *m3* and *m5*, and the distance between *m2* and *m3* must be less than the distance between m3 and m1. We can think of these markers as being points on a straight line. In this case, a minimal spanning tree will link the markers in the proper sequence. However, we can’t precisely know the true recombination frequencies of a set of genetic markers. If we think of the recombination frequencies as being two numbers, the true recombination frequencies plus a noise term, then as the level of “noise” increases the minimal spanning tree is increasingly corrupted by this noise. At a certain low level of noise it is no longer possible to properly order the genetic markers, but it may still be possible to separate the genetic markers that are on different linkage groups. At higher levels of noise, it may not even be possible to correctly separate the genetic markers that are on different linkage groups. There is a commonly used tool, *MSTmap*, which attempts to use information from the minimal spanning tree to construct genetic linkage maps [13]. However the quality of the solutions produced from the minimal spanning tree is highly dependent on the quality and reliability of the recombination frequencies.

By posing genetic linkage map construction as a Traveling Salesperson Problem, we add the additional constraint that we are selecting small weights and imposing an ordering on all of the genetic markers. Methods that only look at the minimal spanning tree do not impose an ordering on all of the genetic markers. By imposing an ordering on all of the genetic markers, we also obtain a solution that is more robust to noise, errors and missing data in the recombination frequencies.

Here we present *TSPmap*: an R package that applies TSP algorithms to the generation of genetic linkage maps from genetic marker data. We compared this method with commonly used tools, *JoinMap* and *MSTmap*, with simulated datasets of varying marker number and missing/erroneous markers, and found that this new tool generates maps in less time and with equal or higher quality.

## Implementation

An important element in our strategy for constructing genetic linkage maps is to exploit problem decomposition. A fundamental motivation for exploiting problem decomposition is to break large problems into smaller problems. This is particularly important if we want to use an exact solver for the TSP. An exact solver might perform reasonably for 2000 markers, but then require an unreasonable amount of time to solve a TSP with 10,000 markers. A natural form of problem decomposition is to separate different groups of markers that are on different chromosomes. For example, if we construct the minimal spanning tree, we may not be able to determine the ordering of all of the genetic markers. However, we may be able to determine that certain groups of genetic markers are on different linkage groups with high probability by examining the minimal spanning tree. In other cases, we may run a heuristic TSP solver, which is less sensitive to problem size, to generate an initial high quality solution. From this initial solution, we may determine that different groups of markers either are on different linkage groups or might be on different linkage groups. By separating the markers into different groups, we can solve each group as a separate instance of a TSP, and then reassemble the solutions for each group into one overall solution. Thus, the proposed solver uses a mix of heuristic methods to generate initial solutions to the TSP instances, and then uses an exact solver on groups of markers that are clearly on the same linkage group.

### TSP solvers

**Fig. 1:**
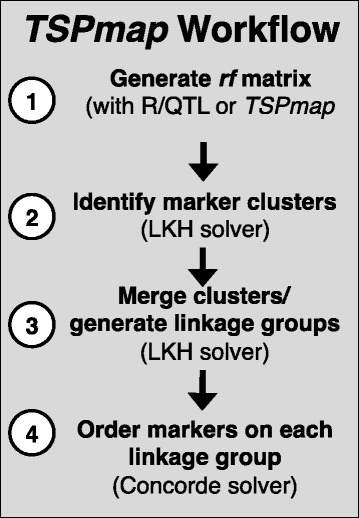
Workflow for *TSPmap* program. First, a matrix of recombination frequencies (*rf*) between markers is generated (either by R/QTL or with functions included with *TSPmap*). Next, the LKH solver is used to identify clusters of markers and merge clusters into linkage groups. Lastly, the Concorde solver is used to correctly order markers in each linkage group

*TSPmap* uses two TSP solvers. Lin-Kernighan-Helsgaun (LKH) is an heuristic local search algorithm based on the Lin-Kernighan algorithm [19, 20]. LKH is able to run in significantly less time than an exact solver. The second is Concorde, an exact solver based on the branch-and-bound method, a technique used to prune the search space and limit unnecessary exploration of areas of the state space which are guaranteed not to produce improvement on an already existing result [18]. Because Concorde is an exact method, it is guaranteed to find the optimal TSP solution. This causes much longer run times than that of LKH, especially as the problem size (number of markers) grows. With *TSPmap*, LKH is used in the early stages of the mapping process, namely to identify and separate the linkage groups. Once these groups are identified, the final order of markers on each linkage group is determined using the exact solution implemented by Concorde (Fig. 1).

### TSPmap algorithm

#### Computation of pair-wise recombination frequency (rf)

*TSPmap* uses a matrix of recombination frequencies (*rf*) between markers to identify linkage groups and order markers. In mapping populations where genotypes are almost entirely homozygous, such as those comprised of recombinant inbred lines (RILS) or generated through double haploidization, *TSPmap* includes a pipeline for quickly generating *rf* matrices. First, genotype data in a matrix format are filtered by removing duplicate and heterozygous markers, as well as markers above a user-defined threshold of similarity across all individuals. Since smaller *rf* values indicate higher correlation between markers, the smaller recombination frequency values are most relevant to the construction of the linkage map. The larger values represent unlinked markers and thus are of limited utility. Because the performance of the TSP solver is slowed by the inclusion of these values, the user may input a cutoff threshold (default set to 0.4) above which all *rf* values are inflated to 0.5, preventing the solver from spending computation time optimizing these non-informative values. However, setting the threshold too low could begin to affect the clustering and the final ordering of markers within the linkage group. This is especially true for noisy datasets where relatively high *rf* values may still be informative.

Missing data can cause recombination frequencies to be underestimated or overestimated, depending on whether the missing call represents a difference or similarity between the two markers. To account for this uncertainty, recombination frequency values for markers with missing data are adjusted to lie at the midpoint of the possible *rf* value range. This prevents the TSP solver from erroneously linking pairs of markers because of missing calls in the data set.

*TSPmap* can also take as input *rf* matrices generated by R/QTL [21] for different types of mapping populations, including those with large numbers of heterozygous markers, such as F_2_ intercrosses and backcrosses. These R/QTL formatted *rf* matrices can then be used for downstream steps in the *TSPmap* algorithm. However, it should be noted that creating *rf* matrices in R/QTL can be time intensive with large numbers of markers, and may slow the overall workflow of generating linkage maps with *TSPmap*.

#### Clustering to form linkage groups

In the typical TSP formulation, the solution is a Hamiltonian cycle. That is, the solution is a tour in which each vertex is visited exactly once and ends at the beginning vertex, thus forming a complete cycle. In the case of a genetic linkage map, we have no need to consider the linkage between the last marker in the linkage group and the first. In fact, allowing the TSP to run in this standard configuration will negatively impact the result, since the algorithm will include the value of this last edge when attempting to minimize the total tour cost, and the *rf* value between the beginning node and ending node are expected to be large since they are on opposite ends on the linkage group. To eliminate this effect we convert TSP problem into a Hamiltonian path problem (HPP). To accomplish this, we introduce into the TSP weight matrix a dummy vertex that has a zero-weight connection to all other vertices. The inclusion of this vertex allows the TSP solver to connect the last vertex in the tour with the first without incurring the large-weight penalty associated with the *rf* value between two distant vertices, in essence allowing the solver to construct a non-cyclic path [22].

Using the recombination frequency matrix, the first step is to connect the unordered dataset into a minimum spanning tree and then break the spanning tree into large clusters that may contain markers from more than one linkage group. This is because we only want to break the spanning tree at a location that has a very high probability of being a transition between chromosomes. To further aid decomposition, the user inputs an initial estimate of the number of linkage groups (chromosomes), *k*, into the algorithm as a parameter. Along with the number of markers, *m*, this is used to define the minimum size of a cluster (*s*) according to the formula

$$ s=\frac{m}{2k} $$

By calculating *s* in this way the algorithm is meant to penalize small clusters, but allow for the possibility of large variation in physical size and marker density among chromosomes. However, we acknowledge that the assigned minimum chromosome size may not hold true in species with extreme variation in chromosome size, e.g. birds and some other vertebrates.

The algorithm begins by constructing the minimum spanning tree and breaking it apart at the largest 1*.*5*k recombination frequency values. If this does not produce *k* significant clusters, the next-largest *rf* value is added to the list of cut points between clusters. This is repeated until at least *k* significant clusters have been produced. In this way, more cuts are made than by simply breaking into the number of known chromosomes, because some chromosomes might not neatly form a single linkage group while other pairs might not have a nice break between linkage groups. Therefore, the algorithm is designed make more cuts, and later test which mergers are best.

**Fig. 2:**
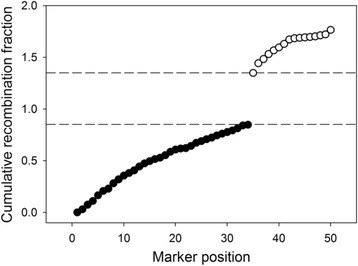
Separation of clusters containing markers from two different linkage groups. If the TSP solution of a cluster shows a large jump in *rf* value between two adjacent markers, this indicates that markers from two different linkage groups are present and the cluster is separated at this point. Clusters are broken into multiple clusters between markers with the largest recombination frequency (dashed lines) to separate markers from different linkage groups (open versus closed circles)

Next, each cluster is run through the approximate TSP solver, LKH, and checked for *rf* values that exceed a user-specified threshold. When such a value is detected, the cluster is broken apart to ensure that the final clusters do not contain markers from more than one linkage group (Fig. 2). This allows the user to control the sensitivity of the linkage group formation by setting what recombination frequency values may exist within a linkage group.

Once the markers have been broken into well-separated clusters, the next step is to merge clusters that belong together as a single linkage group. Clusters are processed in order of increasing size, based on the logic that a small cluster is more likely to be merged with a larger cluster. Each cluster is processed through three stages to determine with which cluster, if any, it should be merged.

**Fig. 3:**
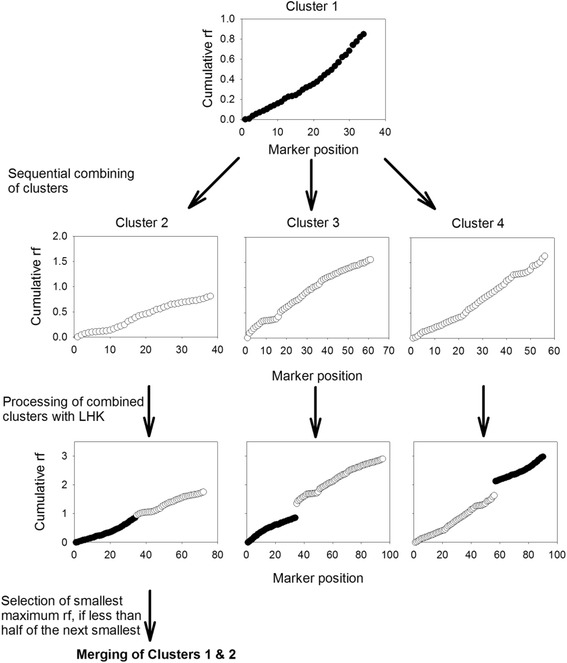
Merging of clusters to create final linkage groups. To create final linkage groups, clusters are sequentially combined, with the resulting recombination frequencies compared to determine clusters to be merged

The first stage involves examination of the *rf* matrix to see if the cluster can be directly merged with another without any further analysis. The second stage involves combining clusters pairwise and processing them with LKH to see if the Hamiltonian path clearly indicates whether the clusters should be merged, based on a merger having a maximum *rf* less than half of the next smallest merger (Fig. 3). Finally, if a cluster does not pass the criterion for being merged in stage 2, the third stage attempts to verify which cluster it belongs to using a reciprocal matching technique of the most likely candidate cluster. If a cluster does not meet any of these criteria, it is considered to be a complete linkage group.

#### Ordering of markers

The final clusters (linkage groups) are processed using Concorde to produce the optimal linkage map for the given data. Because Concorde is an exact solver it is able to find the exact TSP solution to the order of markers in within each linkage group. Finding an exact solution does not guarantee that the order of the markers is absolutely correct; instead, the exact solution is the best solution possible given the available recombination frequencies. Experimental datasets can contain a few markers that are highly erroneous across the entire population. Once TSPmap has determined the marker order, these highly erroneous markers can be identified and dropped using tools available in R/QTL (i.e. droponemarker function) [21] prior to subsequent analyses such as QTL mapping.

### Implementation in R

This procedure was implemented in R, version 3.2.5 [23]. The algorithms that identify duplicate markers and compute the recombination frequency matrix were implemented in C and are called via R’s built-in C interface. The *TSPmap* R package is freely available and includes a user tutorial vignette and example datasets.

### Simulations, comparative mapping algorithms and measures of performance

Datasets representing a mapping population of 300 recombinant inbred lines were simulated using the R/QTL package [21]. Datasets were generated in a factorial design in which each dataset was comprised of a total number of markers (*m*) of 1000 or 4000, evenly distributed across five linkage groups or 10,000 distributed across 10 linkage groups, a genotype error rate (η) of 0.0, 0.01, or 0.05, and a missing genotype rate (γ) of 0.0, 0.05, or 0.10. Five replicates of each configuration were created.

For each simulated marker dataset we generated linkage maps with *TSPmap*. An example script for implementation of *TSPmap* with these data can be found in Additional file 1: File S1. For comparison, datasets were also run on *JoinMap* 4.0 [12] and *MSTmap* [13]. For *JoinMap*, the maximum likelihood algorithm was used, as the regression method gave unreasonably long runtimes for the 4000-marker datasets, and the results for 1000-marker datasets were determined to be higher quality using the maximum likelihood algorithm. Default parameters were used, except values were increased to 5000 for chain length, 10,000 for number of chains without improvement before stopping, and 1000 for chain length per Monte Carlo EM cycle. Additionally, we created linkage maps with these simulated data using *MSTmap*, which uses a minimum spanning tree algorithm [13]. The exact settings used to create linkage maps with simulated datasets in *MSTmap* can be found in Additional file 2: File S2, which is an input file for a simulated dataset into *MSTmap*.

To quantitatively measure the quality of a solution, we compared the final number of linkage groups (chromosomes) *c*, to the true number of chromosomes in the simulated data. Additionally, we calculated the number of erroneous pairs, *E,* in each solution. This number is the number of pairs of markers which appear in reversed order in their estimated positions as compared to their true positions in the simulated data [13].

## Results

### Identification of linkage groups

**Fig. 4:**
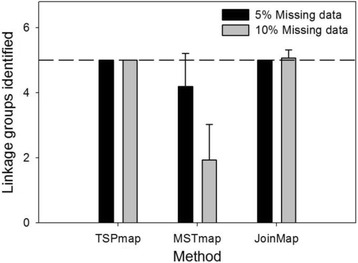
Linkage group identification among the methods. Error bars, SD; dashed line, number of linkage groups in 4000-marker simulated data

*TSPmap* correctly identified the linkage groups for all simulated data sets (Table 1; Fig. 4). In contrast, the performance of *MSTmap* in correctly identifying linkage groups proved sensitive to the number of markers, as well as missing data and genotyping error. The *c* values (number of linkage groups) for *MSTmap* in Table 1 show that multiple linkage groups often remain grouped in the final solution, and this behavior becomes much more pronounced for the 4000-marker data sets. For this reason, *c* values are not reported where *MSTmap* was unable to separate the majority of the linkage groups, as the value is not meaningful. The *c* values in Table 1 are only calculated for datasets where *MSTmap* produced 4 or 5 linkage groups (when 4 linkage groups were produced, the groups were manually separated before computing the value of *E, the number of erroneous marker pairs*). *JoinMap* failed to correctly identify the linkage groups for only one data set and generally performed similarly to *TSPmap*, albeit with much longer run times.

**Table 1:** Comparison of *TSPmap*, *MSTmap*, and *JoinMap*

			TSPmap		*MSTmap*		*JoinMap*	
*n*	*γ*	*η*	*E*	*c*	*E*	*c*	*E*	*c*
1000	0	0	46.2	5	166.4	5	45.8	5
		0.01	60.8	5	196.2	5	55.6	5
		0.05	148.2	5	226.4	5	64.4	5
	0.05	0	62.8	5	169.6	5	60.4	5
		0.01	78.8	5	255.4	5	77.4	5
		0.05	184.4	5	330	4.8	103.4	5
	0.1	0	92	5	178.6	4.8	149.2	5
		0.01	113.8	5	9070	4.4	173.2	5
		0.05	210.8	5	5313.2	4.2	211.4	5
4000	0	0	188.6	5	680.4	4.8	426,090.8	5
		0.01	268.2	5	788.8	4.8	488,497	5
		0.05	596.8	5	875.8	4.6	317,156.8	5
	0.05	0	271.6	5	80,507	4	389,329	5
		0.01	350.2	5	192,508	4	467,511	5
		0.05	698.4	5	1325.8	4.6	529,166.6	5
	0.1	0	364.6	5	639,625	2	711,720.2	5
		0.01	437.6	5	1,166,876	1.6	625,957.8	5
		0.05	826.2	5	927,429	2.2	523,810.4	5.1

*E* is the number of erroneous pairs, *c* is the number of linkage groups produced by each algorithm, *γ* is the percentage of missing data, *η* is the error rate within the data. Five data sets were produced for each parameter combination Reported values are the mean

### Ordering of markers

Ordering of markers, which is both computationally intensive and sensitive to missing and erroneous data, is the limiting step when using NGS data to conduct linkage mapping. To compare the accuracy and efficiency of *TSPmap* with *MSTmap* and *JoinMap*, we tested the number of miss-ordered markers relative to simulated positions across a number of marker dataset sizes, error rates and missing data contents (Table 1). As expected, all three approaches solved the marker-ordering problem effectively in the complete 1000 marker datasets with low error rate. These simulated datasets mimic traditional genotyping techniques and represent easily solvable computational problems. The main difference among methods was computational time, where both *MSTmap* and *TSPmap* dramatically outperformed *JoinMap*. However, as error rate (η) and missing genotype rate (γ) increased, *TSPmap* and *JoinMap* dramatically outperformed *MSTmap*, which exhibited very high E values in the datasets with the most missing and erroneous data. It appeared that linkage group assembly represented the error-prone step in the *MSTmap* protocol, where an inability to fully separate the linkage groups led to substantial marker mis-ordering (Table 1).

**Fig. 5:**
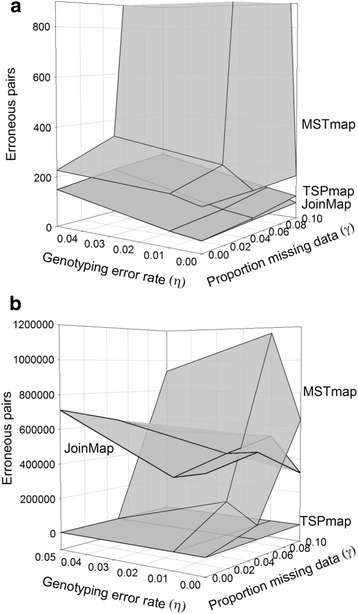
Dependence of erroneous pairs in marker order on missing data and genotyping error. A, 1000-marker datasets; B, 4000-marker datasets

We observed the most dramatic improvements in performance of *TSPmap* among the larger simulated datasets, which are more representative of GBS mapping populations. For 4000-marker datasets, *TSPmap* produced solutions with fewer errors than both *JoinMap* and *MSTmap*. *TSPmap* performed only slightly worse with 4000 markers than with 1000 markers. This is in contrast to both other methods we tested, which were strongly affected by increased marker number (Fig. 5). The likelihood of mis-ordering should scale linearly with the number of markers. For example, in the 4000-marker datasets, we expected 4× higher *E* values than the 1000-marker datasets. Indeed, we found that the overall solution quality from *TSPmap* was generally constant despite the increase in the number of markers. However, this was not the case for *MSTmap* and *JoinMap*, which exhibited exponentially greater E-values in the larger datasets.

**Fig. 6:**
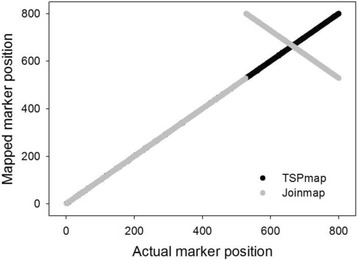
Marker order solutions for an example linkage group from a 4000-marker dataset (*γ* = 0.05, *η* = 0.01). An inversion of part of the linkage group leads to a large *E*-value in the *JoinMap* solution. Black circles, *TSPmap*; grey circles, *JoinMap*

*TSPmap* was the least sensitive to genotyping errors and missing data. Markers ordered by *JoinMap* had substantially higher *E* values (Fig. 5b). This is attributable to its tendency to reverse large segments of the linkage groups with respect to the true solution (Fig. 6). *MSTmap* showed dramatic sensitivity to missing data (Fig. 5).

Finally, *TSPmap* also performed well with 10,000-marker datasets (Table 2), producing results in less than one hour. In contrast, *JoinMap* was computationally limited at 10,000 markers, taking more than 35 h per linkage group. For comparison, *TSPmap* generated maps with 10,000-marker datasets of higher quality than 4000-marker dataset maps generated by both *MSTmap* and *JoinMap*. The dataset comprising 10,000 markers was not analyzed with *MSTmap* because quality scores were already low with 4000 markers (Table 1).

**Table 2:** *TSPmap* results for 10,000-marker datasets

*γ*	*η*	*E*	*c*	Run time (sec)
0	0	495.4	10	1672
	0.01	630.4	10.2	2019
	0.05	1478.4	10	2786
0.05	0	648.8	10	1690
	0.01	824.4	10	1858
	0.05	1724.6	10.2	2134
0.1	0	908	10	2273
	0.01	1116.6	10.2	2260
	0.05	2106.6	10	2265

These datasets contained 10 linkage groups. E is the number of erroneous pairs, c is the number of linkage groups produced by each algorithm, *γ* is the percentage of missing data, *η* is the error rate within the data. Five data sets were produced for each parameter combination. Reported values are the mean

The preceding analyses were performed using simulated genetic marker dataset representing RIL mapping populations only. The efficacy of using recombination frequencies to generate linkage maps can vary depending on the type of mapping population [24, 25]. Therefore, to test the ability of *TSPmap* to create linkage maps with other population types, we evaluated and confirmed the accuracy of *TSPmap* in simulated datasets representing multiple types of mapping populations generated from inbred parents including 4-way cross ((A x B) x (C x D)), F_2_, and backcross. We simulated five sets of 27 genotype matrices for each cross type, representing three error probabilities (0, 0.1 and 1%), three missing data probabilities (0, 0.1, and 1%) and three marker densities (2 cM, 0.5 cM, 0.2 cM). Partially informative (e.g. dominant) markers were not simulated, because recombination fractions cannot be calculated among pairs of markers in 4-way mapping populations that are only informative in alternative crosses. Marker positions of each genotype matrix were randomized, recombination fractions were calculated in *R/qtl*, and the resultant matrix was fed into *TSPmap*. For each dataset, the correlation coefficient between the true marker order and the simulated marker order generated by *TSPmap* was >0.999 (Additional file 3: Figure S1). While the overall order nearly perfect, performance did vary slightly among different mapping populations. Backcross populations, which have the least recombination and only two alleles, was more prone to slightly incorrect orders among the high error and high missing data simulations. However, both 4-way and F_2_ population orders were more accurate than the RIL, indicating that our focus on RIL populations represents a conservative estimate of the accuracy of TSPmap.

To examine the performance of *TSPmap* with experimentally generated marker data, we created maps using previously published marker data from RIL populations of *Arabidopsis thaliana* [26] and rice [27]. Although the true solution cannot be known for such experimental datasets, we observe that the *TSPmap* linkage map solution generally matched that of *JoinMap* (Additional file 4: Figure S2). Given the results of our analyses with simulated datasets, those differences between the *TSPmap* and JoinMap solutions that are observed (ie. Additional file 4: Figure S2d) may reflect *TSPmap*’s greater accuracy when creating maps from larger datasets containing higher levels of missing and erroneous data, as is often encountered in experimental data.

## Conclusions

Genotyping by sequencing is now the preferred method to genotype mapping populations. However, tools to process and order these error prone and incomplete datasets remain undeveloped. Here we confirm that current widely used genetic mapping software does not produce sufficiently accurate genetic maps from datasets of the size generated by NGS. We offer a solution through an implementation of the Travelling Salesperson Problem for generation of genetic maps, *TSPmap*, which produces low-error maps even with large datasets and high rates of missing and erroneous marker calls. When compared to other benchmark methods of linkage mapping, *JoinMap* and *MSTmap*, *TSPmap* was faster (Table 2) and far more accurate (Table 1, Fig. 5). Additionally, *TSPmap* proved robust for analysis of large marker datasets, performing well even with 10,000 markers (Table 2). We acknowledge that the performance of *JoinMap* to generate linkage maps in the simulations we present here may be improved by fine-tuning its algorithm parameters; however, *JoinMap*’s long run-times are prohibitive to such exploratory analyses. We also find that *TSPmap* can create accurate linkage maps from simulated marker datasets representing F_2_, backcross, and 4-way mapping populations (Additional file 3: Figure S1), and with experimental marker data (Additional file 4: Figure S2).

## Discussion

In addition to handling large marker datasets efficiently and effectively, *TSPmap* is open source software, increasing its accessibility and flexibility. As a package written in the statistical language R, it is freely available online and users can tailor it to their specific needs. Included with the package is a vignette that will guide users through the functions and workflow of *TSPmap*.

*TSPmap* requires that users indicate the number of expected linkage groups, so it is valuable to know the chromosome count of the species being studied. It would be possible to carefully explore a set of possible *k* values and choose the best fit to the data, in the case that an approximate chromosome number is unknown, but *TSPmap* is highly biased toward the prior, the user-specified number of linkage groups. While we expect that chromosome number will be known for most species in which linkage maps would be created, we note that *TSPmap* may calculate an erroneous number of linkage groups if used with a species of unknown chromosome count.

Since *TSPmap* operates on a recombination fraction matrix, our approach is potentially extendable to any mapping population in which recombination fractions can be calculated, including inbred and outbred breeding designs that are genotyped by either codominant or dominant markers. However, since our simulations have been conducted in *R/qtl* we limit our inference here to inbred-parent breeding designs supported by that environment, including: recombinant inbred, F _2_ , backcross, and intercrossed 4-way phase known populations.

Genetic linkage maps have evolved from calculating linkage disequilibrium between a handful of phenotypic markers [28] to datasets of containing tens of thousands of genomic polymorphisms. With the advancement of low cost sequencing technologies, the number of markers used in the generation of genetic maps is expected to continue to rise. *TSPmap* will be a powerful tool to handle such large datasets into the future, quickly producing high quality maps using a large number of genomic markers.

## Additional files

Additional file 1: Example script for generating linkage maps with *TSPmap*. (R 2 kb)

Additional file 2: Example parameters for generating linkage maps with *MSTmap*. (TXT 593 kb)

Additional file 3: Figure S1. Performance of *TSPmap* with simulated datasets of different types of mapping populations (4way – four-way cross, bc – backcross, f2 – F_2_ population, riself – recombinant inbred lines), marker number (100, 400, 1000), proportions of missing data (0, 0.001, 0.01) and genotyping error rates (0, 0.001, 0.01). The accuracy of the *TSPmap* solution was measured by the correlation coefficient between the true marker order and marker order generated by *TSPmap* for each simulated dataset. Note the scale of the y-axis is 0.99935–1.000. (DOCX 40 kb)

Additional file 4: Figure S2. Linkage maps generated by *TSPmap* using marker datasets from A. *Arabidopsis thaliana* [26] and B. & C. rice [27] compared to those generated by *JoinMap*. (DOCX 213 kb)

## Abbreviations

c: Final number of linkage groups
E: Number of erroneous pairs
G(V,E): All of the vertices of a graph
GBS: Genotyping-by-sequencing
HPP: Hamiltonian path problem
k: Initial estimate of the number of linkage groups
LKH: Lin-Kernighan-Helsgaun
m: Number of markers
MST: Minimal spanning tree
O(V + E): Polynomial time complexity
QTL: Quantitative trait loci
rf: Recombination frequencies
RILS: Recombinant inbred lines
s: Minimum size of a cluster
TSP: Travelling salesperson problem
γ: Missing genotype rate
η: Genotype error rate

## References

[1] Sun Z, Wang Z, Tu J, Zhang J, Yu F, PBE MV (2007). An ultradense genetic recombination map for Brassica Napus, consisting of 13551 SRAP markers. Theor Appl Genet.

[2] Li W, Zhang J, Mou Y, Geng J, McVetty PBE, Hu S (2011). Integration of Solexa sequences on an ultradense genetic map in Brassica Rapa L. BMC Genomics.

[3] Bowers JE, Bachlava E, Brunick RL, Rieseberg LH, Knapp SJ, Burke JM (2012). Development of a 10,000 locus genetic map of the sunflower genome based on multiple crosses. G3.

[4] Truco MJ, Ashrafi H, Kozik A, van Leeuwen H, Bowers J, Reyes Chin Wo S (2013). An ultra high-density, transcript-based, genetic map of lettuce. G3.

[5] Qiu G-F, Xiong L-W, Han Z-K, Liu Z-Q, Feng J-B, Wu X-G (2017). A second generation SNP and SSR integrated linkage map and QTL mapping for the Chinese mitten crab Eriocheir Sinensis. Sci Rep.

[6] Yan J, Yang X, Shah T, Sánchez-Villeda H, Li J, Warburton M (2010). High-throughput SNP genotyping with the Goldengate assay in maize. Mol Breed.

[7] Elshire RJ, Glaubitz JC, Sun Q, Poland JA, Kawamoto K, Buckler ES (2011). A robust, simple genotyping-by-sequencing (GBS) approach for high diversity species. PLoS One.

[8] Semagn K, Babu R, Hearne S, Olsen M (2014). Single nucleotide polymorphism genotyping using Kompetitive allele specific PCR (KASP): overview of the technology and its application in crop improvement. Mol Breed.

[9] Lander ES, Green P (1987). Construction of multilocus genetic linkage maps in humans. Proc Natl Acad Sci U S A.

[10] Stam P (1993). Construction of integrated genetic linkage maps by means of a new computer package: JoinMap. Plant J.

[11] Schiex T, Gaspin C (1997). CARTHAGENE: constructing and joining maximum likelihood genetic maps. Proc Int Conf Intell Syst Mol Biol.

[12] Van Ooijen JW (2006). JoinMap 4: software for the calculation of genetic linkage maps in experimental populations.

[13] Wu Y, Bhat PR, Close TJ, Lonardi S (2008). Efficient and accurate construction of genetic linkage maps from the minimum spanning tree of a graph. PLoS Genet.

[14] Cheema J, Dicks J (2009). Computational approaches and software tools for genetic linkage map estimation in plants. Brief Bioinform.

[15] Mester D, Ronin Y, Minkov D, Nevo E, Korol A (2003). Constructing large-scale genetic maps using an evolutionary strategy algorithm. Genetics.

[16] Iwata H, Ninomiya S (2006). AntMap: constructing genetic linkage maps using an ant Colony optimization algorithm. Breed Sci.

[17] Van Ooijen JW, Jansen J (2013). Genetic mapping in experimental populations.

[18] Applegate D, Bixby R, Chvátal V, Cook W (2006). Concorde TSP solver.

[19] Lin S, Kernighan BW (1973). An effective heuristic algorithm for the traveling-salesman problem. Oper Res.

[20] Helsgaun K (2000). An effective implementation of the Lin-Kernighan traveling salesman heuristic. Eur J Oper Res.

[21] Broman KW, Wu H, Sen Ś, Churchill GA (2003). R/QTL: QTL mapping in experimental crosses. Bioinformatics.

[22] Thompson GL, Singhal S (1985). A successful Hamiltonian algorithm for the undirected path problem. Discret Appl Math.

[23] Team RC (2016). R: a language and environment for statistical computing.

[24] Maliepaard C, Jansen J, Van Ooijen JW (1997). Linkage analysis in a full-sib family of an outbreeding plant species: overview and consequences for applications. Genet Res.

[25] Preedy KF, Hackett CA (2016). A rapid marker ordering approach for high-density genetic linkage maps in experimental autotetraploid populations using multidimensional scaling. Theor Appl Genet.

[26] Ågren JA, Oakley C, McKay JK, Lovell JT, Schemske DW (2013). Genetic mapping of adaptation reveals fitness tradeoffs in Arabidopsis Thaliana. PNAS.

[27] Tanger P, Klassen S, Mojica JP, Lovell JT, Moyers BT, Baraoidan M, Naredo NEB, McNally KL, Poland J, Bush DR, Leung H, Leach JE, McKay JK (2017). Field-based high throughput phenotyping rapidly identifies genomic regions controlling yield components in rice. Sci Rep.

[28] Sturtevant AH (1913). The linear arrangement of six sex-linked factors in drosophila, as shown by their mode of association. J Exp Zool.

[29] Wickham H, Chang W (2016). devtools: tools to make developing R packages easier.

