# Supplementary material for: TSPmap, a tool making use of traveling salesperson problem solvers in the efficient and accurate construction of high-density genetic linkage maps
Source: BioData Min. 2017 Dec 19;10:38. doi: 10.1186/s13040-017-0158-0 (PMC5735504; doi:10.1186/s13040-017-0158-0)
Supplement: Supplementary file 3 — Performance of TSPmap with simulated datasets of different types of mapping populations (4way – four-way cross, bc – backcross, f2 – F2 population, riself – recombinant inbred lines), marker number (100, 400, 1000), proportions of missing data (0, 0.001, 0.01) and genotyping error rates (0, 0.001, 0.01). The accuracy of the TSPmap solution was measured by the correlation coefficient between the true marker order and marker order generated by TSPmap for each simulated dataset. Note the scale of the y-axis is 0.99935–1.000. (DOCX 40 kb) [file 13040_2017_158_MOESM3_ESM.docx]

**Figure S1.** Performance of *TSPmap* with simulated datasets of different types of mapping populations (4way – four-way cross, bc – backcross, f2 – F_2_ population, riself – recombinant inbred lines), marker number (100, 400, 1000), proportions of missing data (0, 0.001, 0.01) and genotyping error rates (0, 0.001, 0.01). The accuracy of the *TSPmap* solution was measured by the correlation coefficient between the true marker order and marker order generated by *TSPmap* for each simulated dataset. Note the scale of the y-axis is 0.99935–1.000.
