# Supplementary material for: TSPmap, a tool making use of traveling salesperson problem solvers in the efficient and accurate construction of high-density genetic linkage maps
Source: BioData Min. 2017 Dec 19;10:38. doi: 10.1186/s13040-017-0158-0 (PMC5735504; doi:10.1186/s13040-017-0158-0)
Supplement: Supplementary file 4 — Linkage maps generated by TSPmap using marker datasets from A. Arabidopsis thaliana [26] and B. & C. rice [27] compared to those generated by JoinMap. (DOCX 213 kb) [file 13040_2017_158_MOESM4_ESM.docx]

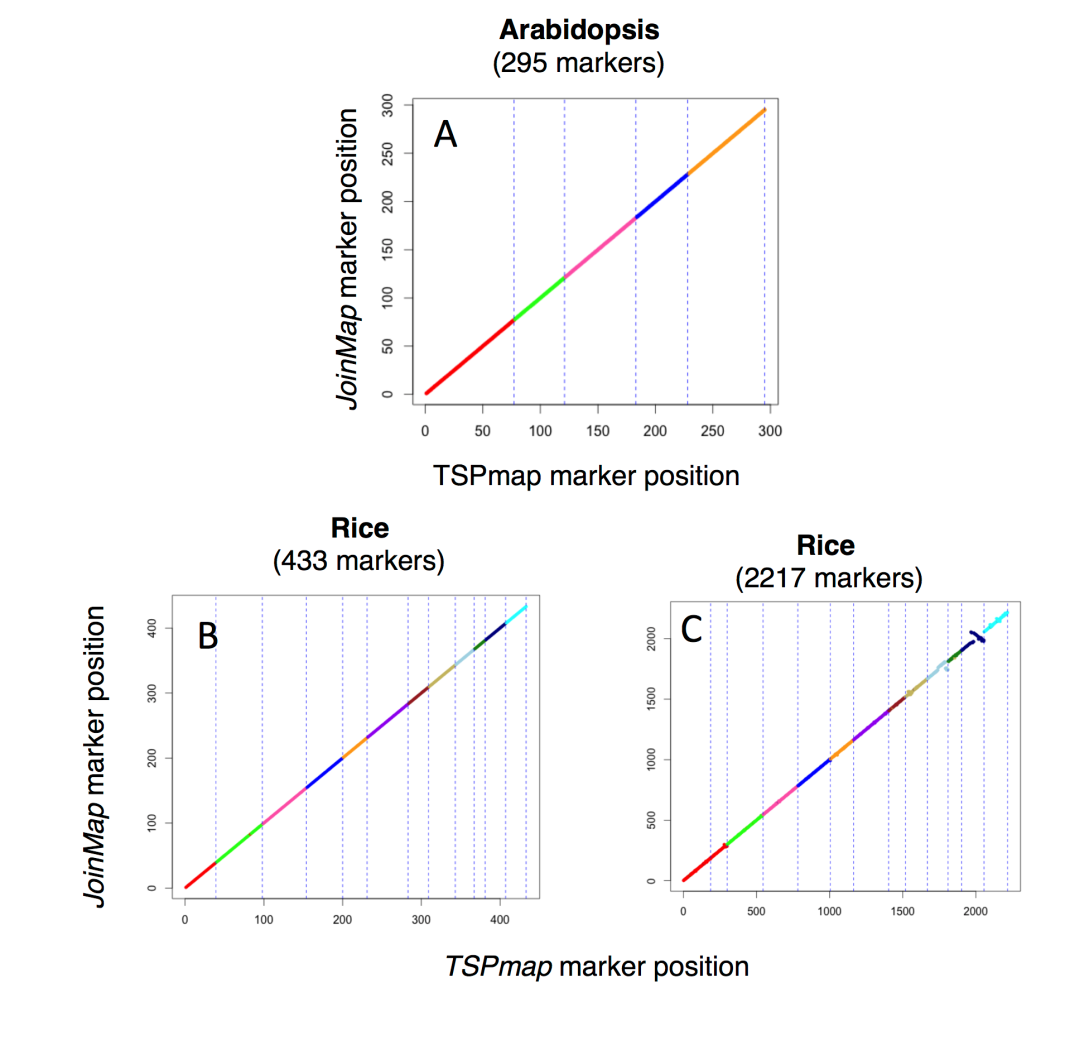


**Figure S2**. Linkage maps generated by *TSPmap* using marker datasets from A. *Arabidopsis thaliana* [26] and B. & C. rice [27] compared to those generated by *JoinMap*.
